# Electromagnetic energy density in hyperbolic metamaterials

**DOI:** 10.1038/s41598-022-14909-0

**Published:** 2022-06-24

**Authors:** Afshin Moradi, Pi-Gang Luan

**Affiliations:** 1 Department of Engineering Physics, Kermanshah University of Technology, Kermanshah, Iran; 2 Department of Optics and Photonics, National Central University, Jhongli District, Taoyuan City , 320317 Taiwan

**Keywords:** Metamaterials, Electrical and electronic engineering

## Abstract

We present the theory of electromagnetic energy propagation through a dispersive and absorbing hyperbolic metamaterial (HMM). In this way, the permittivity tensor components of HMM (especially, nanowire HMM) may appear to be hopeless, but as a non-trivial step, we find that they can be cast into more transparent forms. We find under the influence of an electromagnetic wave, the responses of nanowire HMM (multilayer HMM) in the directions perpendicular to and parallel to the optical axis are similar to those of Lorentz (Drude) and Drude (Lorentz) media, respectively. We obtain simple expressions for the electromagnetic energy density formula of both typical structures of HMMs, i.e., nanowire and multilayer HMMs. Numerical examples reveal the general characteristics of the direction-dependent energy storage capacity of both nanowire and multilayer HMMs. The results of this study may shed more physical insight into the optical characteristics of HMMs.

## Introduction

Metamaterials are artificial media that usually refer to arrays of wires and split-ring resonators (SRRs)^1,2^. They have unusual optical properties such as negative refractive index^3^, subwavelength imaging^4^, and indefinite permittivity^5^. A fundamental problem concerning dispersive and absorbing metamaterials is how to obtain electromagnetic energy density^6–11^. Actually, the contradictory results of electromagnetic energy density in lossy wire-SRR metamaterials have been the subject of theoretical controversy^12–15^.

If the losses are negligible, one can correctly obtain the electromagnetic energy density of such media by using the well-known Landau formula^16^. In the presence of damping effects, on the other hand, difficulties arise in attempting to calculate the energy associated with an electromagnetic wave passing through the wire-SRR metamaterials. During past years, several researchers achieved different results in different ways^12–15^. The question then becomes, what is the correct result for the electromagnetic energy density in dispersive and absorbing wire-SRR metamaterials? In this way, one of the present authors derived the electromagnetic energy density formula, which is consistent with the Landau formula, when the losses are negligible^17^. Then, Luan *et al.* obtained the electromagnetic energy density formula for the single-resonance chiral metamaterials^18^, using the same approach discussed in^17^.

An HMM refers to an extremely anisotropic uniaxial optical media and has received much attention recently^19–25,28^. This medium has opposite signs of the two principal values of the permittivity tensor along and perpendicular to its optical axis. Mathematically, the dispersion of an electromagnetic wave in such a medium has a hyperbolic shape. By definition, there are two types of HMMs. Type I with a predominantly dielectric nature, and type II with a predominant metallic behavior^26^. Typically, HMMs contain alternating deeply subwavelength metal and dielectric features and there are different configurations of HMMs such as metallic nanowire structures and dielectric-metal multilayer structures. Note that the signs of the two effective permittivities of such structures are frequency-dependent, so they are not definitely type-I HMM or type-II HMM and therefore an HMM is well-known as an indefinite medium^27^. However, a periodic array of metallic nanowires (for example gold nanowires) embedded in a host dielectric matrix (for example $\hbox {SiO}_{2}$ ) may be an example of type-I HMM in the visible wavelength and near-IR range^28^. Also, a multilayer medium consisting of dielectric and metallic layers (for example gold-$\hbox {SiO}_{{2}}$ HMM) may be an example of type-II HMM in the visible and near-IR range^28^. There are quite a number of interesting works on the characteristics and applications of HMMs, such as directional transmission^29,30^, long-range interaction^31,32^, and super-resolution imaging^33,34^. Also, the resonant modes of HMMs with oscillators have been studied in waveguide structures^35^ and cavity structures^36,37^. Interestingly, more recently, it has been shown that the propagation of sound waves in HMMs is similar to the gravitational waves, which means the quantized sound waves (phonons) are similar to gravitons^38^.

But what is the electromagnetic energy formula for a lossy HMM? Recently, one of the present authors studied the propagation of electromagnetic energy in a lossy multilayer HMM for a simple case^39^. However, the electromagnetic energy density formula in a lossy nanowire HMM has not been derived yet, perhaps because of its difficulty. In fact, the effective permittivities of HMMs are more complex than the wire-SRR and chiral metamaterials.

Solving the above-mentioned problem is the main motivation of the present work. However, to do this, the components of the well-known effective permittivity tensor of a nanowire HMM may appear to be hopeless, but they can be cast into more transparent forms. Actually, as a non-trivial and key step, we obtain familiar forms for the effective permittivities of nanowire HMMs and multilayer HMMs. Then, we can easily derive the energy density associated with an electromagnetic wave passing through them. Note that the new appropriate forms for the effective permittivities of nanowire HMMs and multilayer HMMs may shed more physical insight into the optical characteristics of HMMs. Finally, for the sake of completeness, we also find a new transparent form for the effective permittivity of a composite of metallic nano-spheres embedded in a host matrix (see the  Supplementary Material).

## Theory

Let us consider a dispersive and absorbing HMM as an effective uniaxial crystal. In the presence of an oscillating electric field $\mathbf{E }=\mathbf{E }_{\parallel }+\mathbf{E }_{\bot }$ of frequency $\omega$ and long-wavelength vibration, the equation of motion of this medium may be represented by the Lorentz equation. We have^40^

1 $$\begin{aligned} m\ddot{\mathbf{r }}_{\vartheta }+m\gamma \dot{\mathbf{r }}_{\vartheta } +m\omega _{\mathrm {0}\vartheta }^{2}\mathbf{r }_{\vartheta }=q\mathbf{E }_{\vartheta } , \end{aligned}$$

with $\vartheta =\parallel ,\bot$. Also, *q* is the electric charge, *m* is the effective mass of each electric charge, $\mathbf{r }=\mathbf{r }_{\parallel }+\mathbf{r }_{\bot }$ is the displacement of the oscillators, $\omega _{\mathrm {0}\vartheta }$ is the resonance frequency of the charges and $\gamma$ is the damping frequency. Note that the *z*-axis is along the optical axis of the HMM and $E_{\parallel }=E_{z}$.

Suppose that there are *N* oscillators in the HMM with volume *V* and let us represent the effect of the high resonances by real constant background dielectric constants $\varepsilon _{\infty \parallel }$ and $\varepsilon _{\infty \bot }$. Then, we have

2 $$\begin{aligned} \mathbf{P }_{\vartheta }=\dfrac{Nq}{V}\mathbf{r }_{\vartheta }+\varepsilon _{0}\left( \varepsilon _{\infty \vartheta }-1 \right) \mathbf{E }_{\vartheta } , \end{aligned}$$

where $\mathbf{P }_{\vartheta }$ is the $\vartheta$ component of complex polarization that has a part due to the oscillators of natural frequency $\omega _{\mathrm {0}\vartheta }$, and a part due to the higher frequency resonances. Therefore, the components of the relative permittivity tensor can be determined by using Eqs. (1) and (2) as $\varepsilon _{\vartheta }=1+P_{\vartheta }/\varepsilon _{0}E_{\vartheta }$. The final outcome is

3 $$\begin{aligned} \varepsilon _{\vartheta }=\varepsilon _{\infty \vartheta }\left( 1-\dfrac{F_{\vartheta }\omega _{0\vartheta }^{2}}{\omega (\omega +i\gamma )-\omega _{0\vartheta }^{2}}\right) , \end{aligned}$$

where $F_{\vartheta }=\omega _{\mathrm {p}\vartheta }^{2}/\omega _{0\vartheta }^{2}$ measures the strength of the HMM resonance, $\varepsilon _{x}=\varepsilon _{y}=\varepsilon _{\bot }$, $\varepsilon _{z}=\varepsilon _{\parallel }$, and $\omega _{\mathrm {p}\vartheta }=\left( Nq^{2}/\varepsilon _{0}\varepsilon _{\infty \vartheta }mV \right) ^{1/2}$.

Indeed, Eq. (3) suggests that the Lorentz formula is a general and suitable form for the components of the relative permittivity tensor of an HMM. The electromagnetic energy density for a Lorentz type of isotropic media [see Eq. (3)] has been derived earlier by Loudon^40^. Thus, the time-averaged total energy density associated with a harmonic wave in a Lorentz type of HMM is

4 $$\begin{aligned} U=U_{\mathrm {EM}}+U_{\mathrm {K}}+U_{\mathrm {P}}, \end{aligned}$$

where

5 $$\begin{aligned} U_{\mathrm {EM}}=\dfrac{1}{4}\left[ \varepsilon _{0}\varepsilon _{\infty \parallel }\vert \mathbf{E }_{\parallel }\vert ^{2}+\varepsilon _{0}\varepsilon _{\infty \bot }\vert \mathbf{E }_{\bot }\vert ^{2}+\mu _{0} \vert \mathbf{H }\vert ^{2}\right] , \end{aligned}$$

is the electromagnetic energy density ($\varepsilon _{0}$ and $\mu _{0}$ are permittivity and permeability of free space, respectively, and $\mathbf{H }$ is magnetic field), and

6 $$\begin{aligned} U_{\mathrm {K}}=\dfrac{1}{4}\dfrac{Nm}{V}\left( \vert \dot{\mathbf{r }}_{\parallel }\vert ^{2}+\vert \dot{\mathbf{r }}_{\bot }\vert ^{2} \right) , \end{aligned}$$

is the kinetic energy density of HMM, and

7 $$\begin{aligned} U_{\mathrm {P}}=\dfrac{1}{4}\dfrac{Nm}{V}\left( \omega _{0\parallel }^{2}\vert \mathbf{r }_{\parallel }\vert ^{2}+\omega _{0\bot }^{2}\vert \mathbf{r }_{\bot }\vert ^{2}\right) . \end{aligned}$$

is the potential energy density of HMM. Therefore, in general, the total energy includes two parts: the first part from the electric and magnetic fields themselves, the second part from the medium response, i.e., the kinetic and potential energies of the charges under the influence of the electromagnetic wave^41^. Also, the time-averaged power loss density can be written as

8 $$\begin{aligned} P_{\mathrm {loss}}=\dfrac{Nm\gamma }{2V}\left( \vert \dot{\mathbf{r }}_{\parallel }\vert ^{2}+ \vert \dot{\mathbf{r }}_{\bot }\vert ^{2}\right) =2\gamma U_{\mathrm {K}} . \end{aligned}$$

Using Eq. (1) to eliminate $\mathbf{r }_{\parallel }$ and $\mathbf{r }_{\bot }$ from Eqs. (5)–(8), we obtain

9 $$\begin{aligned} U=\dfrac{1}{4}\left[ \varepsilon _{0}\Xi _{\parallel } \vert \mathbf{E }_{\parallel }\vert ^{2}+\varepsilon _{0}\Xi _{\bot } \vert \mathbf{E }_{\bot }\vert ^{2}+\mu _{0} \vert \mathbf{H }\vert ^{2}\right] , \end{aligned}$$

where $\Xi _{\parallel }$ and $\Xi _{\bot }$ are the effective energy coefficients as

**Figure 1:**
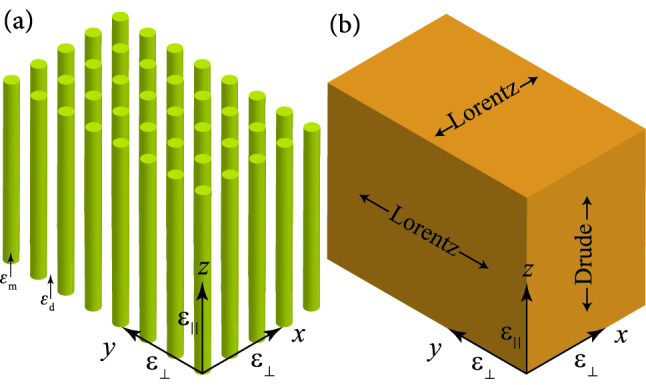
(**a**) Schematic of a periodic array of metallic nanowires embedded in a host dielectric matrix, as a typical 2D geometry of HMMs. The host matrix is not shown. This array of nanowires aligned with the *z*-axis and arranged on a square lattice in the *xy*-plane. (**b**) A nanowire HMM is an effective medium that has different dynamical properties in the direction parallel and perpendicular to its optical axis (the *z*-axis).

10 $$\begin{aligned} \Xi _{\vartheta }=\varepsilon _{\infty \vartheta }\left( 1+\dfrac{F_{\vartheta }\omega _{0\vartheta }^{2}\left( \omega ^{2}+\omega _{0\vartheta }^{2}\right) }{\left( \omega ^{2}-\omega _{0\vartheta }^{2}\right) ^{2} +\gamma ^{2}\omega ^{2}} \right) , \end{aligned}$$

and

11 $$\begin{aligned} P_{\mathrm {loss}}=\dfrac{\gamma \varepsilon _{0}}{2}\left[ \Gamma _{\parallel } \vert \mathbf{E }_{\parallel }\vert ^{2}+\Gamma _{\bot } \vert \mathbf{E }_{\bot }\vert ^{2}\right] =\dfrac{\omega \varepsilon _{0}}{2}{\text {Im}}\left[ \varepsilon _{\parallel } \vert \mathbf{E }_{\parallel }\vert ^{2}+\varepsilon _{\bot } \vert \mathbf{E }_{\bot }\vert ^{2}\right] , \end{aligned}$$

where ${\text {Im}}$ means imaginary part, and $\Gamma _{\parallel }$ and $\Gamma _{\bot }$ are the effective energy loss coefficients as

12 $$\begin{aligned} \Gamma _{\vartheta }=\varepsilon _{\infty \vartheta }\dfrac{F_{\vartheta } \omega _{0\vartheta }^{2}\omega ^{2}}{\left( \omega ^{2}-\omega _{0\vartheta }^{2}\right) ^{2} +\gamma ^{2}\omega ^{2}}. \end{aligned}$$

If the losses are negligible, the time-averaged electromagnetic energy density, for a monochromatic (single frequency) electromagnetic field, is^16^

13 $$\begin{aligned} U=\dfrac{\varepsilon _{0}}{4}\left[ \dfrac{d (\omega \varepsilon _{\parallel }) }{d\omega } \vert \mathbf{E }_{\parallel }\vert ^{2}+\dfrac{d (\omega \varepsilon _{\bot }) }{d\omega } \vert \mathbf{E }_{\bot }\vert ^{2}\right] +\dfrac{\mu _{0}}{4} \vert \mathbf{H }\vert ^{2}. \end{aligned}$$

Setting $\gamma =0$ in Eq. (3) and using Eq. (13), we obtain

14 $$\begin{aligned} U&=\dfrac{1}{4}\varepsilon _{0}\varepsilon _{\infty \parallel }\left( 1+\dfrac{F_{\parallel }\omega _{0\parallel }^{2}\left( \omega ^{2}+\omega _{0\parallel }^{2}\right) }{\left( \omega ^{2}-\omega _{0\parallel }^{2}\right) ^{2} } \right) \vert \mathbf{E }_{\parallel }\vert ^{2}\nonumber \\&\quad +\dfrac{1}{4}\varepsilon _{0}\varepsilon _ {\infty \bot }\left( 1+\dfrac{F_{\bot }\omega _{0\bot }^{2}\left( \omega ^{2}+\omega _{0\bot }^{2}\right) }{\left( \omega ^{2}-\omega _{0\bot }^{2}\right) ^{2} } \right) \vert \mathbf{E }_{\bot }\vert ^{2}+\dfrac{1}{4}\mu _{0} \vert \mathbf{H }\vert ^{2}, \end{aligned}$$

that is exactly the same result as that obtained by setting $\gamma =0$ in Eq. (9). This equality is the first verification of the presented results.

### Nanowire hyperbolic metamaterials

Now, let us consider a periodic array of metallic nanowires with axes parallel to *z*-axis embedded in a host dielectric matrix with the dielectric constant $\varepsilon _{\mathrm {d}}$, as shown in Fig. 1a. The *z*-axis is along the optical axis. Let *f* be the filling fraction of the metallic nanowires in a unit cell satisfying $0<f<1$. This periodic array can be used to construct an electric HMM of type I (in an appropriate frequency region), with the components of effective permittivity tensor given by^21,42^

**Figure 2:**
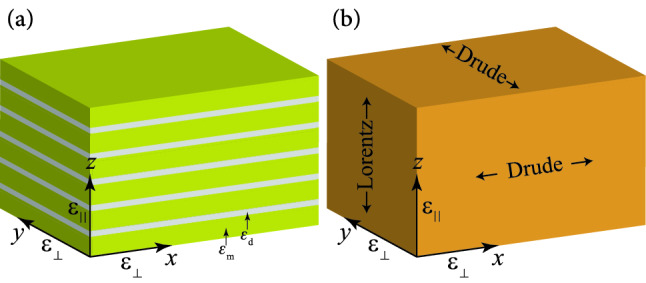
(**a**) Schematic of a multilayer metal-dielectric structure parallel to the *xy*-plane, as a typical 1D geometry of HMMs. (**b**) A multilayer HMM is an effective medium that has different dynamical properties in the direction parallel and perpendicular to its optical axis (the *z*-axis).

15 $$\begin{aligned} \varepsilon _{\mathrm {\parallel }}= & {} f\varepsilon _{\mathrm {m}}+(1-f)\varepsilon _{\mathrm {d}} ,\quad (<0), \end{aligned}$$

16 $$\begin{aligned} \varepsilon _{\bot } & = \varepsilon _{\mathrm {d}}\dfrac{(1+f)\varepsilon _{\mathrm {m}} +(1-f)\varepsilon _{\mathrm {d}}}{(1-f)\varepsilon _{\mathrm {m}} +(1+f)\varepsilon _{\mathrm {d}}}\;,\;\;\;\;\;(>0), \end{aligned}$$

where

17 $$\begin{aligned} \varepsilon _{\mathrm {m}}=\varepsilon _{\infty }\left( 1-\dfrac{\omega _{\mathrm {p}0}^{2}}{\omega \left( \omega +i\gamma \right) }\right) , \end{aligned}$$

shows the relative permittivity of a metallic nanowire with $\varepsilon _{\infty }$ that is the permittivity in high-frequency and $\omega _{\mathrm {p}0}$ as the electron plasma frequency. Here we have relaxed the restrictions in the previous investigation^39^ by considering $\varepsilon _{\infty }$ in Eq. (17). Therefore the formulas might provide more realistic applications.

At this stage, as a non-trivial step, the above effective permittivities can be written as Eq. (3). The recipe for reducing Eqs. (15) and (16) to Eq. (3) is as follows

1. First, replace $\varepsilon _{\mathrm {m}}$ with $\varepsilon _{\infty }$.
2. Replace $\varepsilon _{\vartheta }$ with $\varepsilon _{\infty \vartheta }$ to find $\varepsilon _{\infty \vartheta }$.
3. Find $\omega _{\mathrm {p}\vartheta }^{2}=F_{\vartheta }\omega _{0\vartheta }^{2}$ from $\varepsilon _{\vartheta }<0$, as $\omega _{\mathrm {p}\vartheta }^{2}=f\varepsilon _{\infty }\omega _{\mathrm {p}0}^{2}/\varepsilon _{\vartheta }$ and consider $\omega _{0\vartheta }=0$.
4. Consider $\xi$ as the denominator of $\varepsilon _{\vartheta }>0$, and find $\omega _{0\vartheta }^{2}=(1-f)\varepsilon _{\infty }\omega _{\mathrm {p}0}^{2}/\xi$.
5. Consider $\zeta$ as the product of the denominator and numerator of $\varepsilon _{\vartheta }>0$, and find $\omega _{\mathrm {p}\vartheta }^{2}=\eta ^{2}f\varepsilon _{\mathrm {d}}^{2}\varepsilon _{\infty }\omega _{\mathrm {p}0}^{2}/\zeta$. Note that $\eta$ is a geometrical factor. $\eta =1,2$ and 3 for slab, cylinder, and sphere inclusions, respectively.
6. Finally, for $\varepsilon _{\vartheta }>0$ find $F_{\vartheta }=\omega _{\mathrm {p}\vartheta }^{2}/\omega _{0\vartheta }^{2}$.

Using the mentioned step-by-step procedure, we obtain

**Figure 3:**
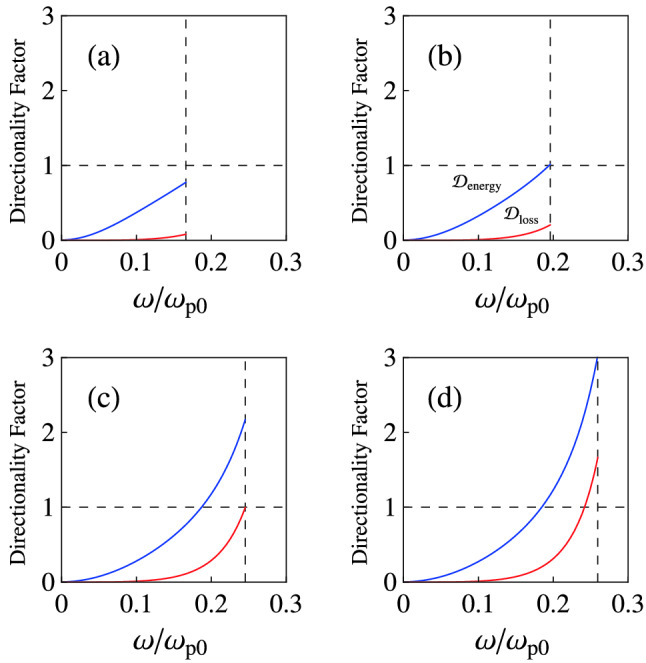
Variation of ${\mathcal {D}}_{\mathrm {energy}}$ (blue curves), and ${\mathcal {D}}_{\mathrm {loss}}$ (red curves) as a function of the dimensionless frequency $\omega /\omega _{\mathrm {p}0}$ for a nanowire HMM as type-I HHM (for example a gold nanowire HMM in the visible and near-IR range^28^). Here, the permittivity $\varepsilon _{\mathrm {m}}$ of the metal is characterized by the Drude model and for the dielectric we use SiO$_{2}$ with $\varepsilon _{\mathrm {d}}=\varepsilon _{\mathrm {SiO_{2}}}=3.9$. For the other parameters we consider $\varepsilon _{\infty }=1$, and $\gamma =0.01\omega _{\mathrm {p}0}$. The vertical dashed line shows $\omega =\sqrt{\omega _{\mathrm {p}\parallel }^{2} -\gamma ^{2}}$. The different panels refer to (**a**) $f=0.1$, (**b**) $f=0.135$, (**c**) $f=0.2$, and (**d**) $f=0.22$.

18 $$\begin{aligned} \varepsilon _{\infty \parallel } & = f\varepsilon _{\infty }+(1-f)\varepsilon _{\mathrm {d}}, \end{aligned}$$

19 $$\begin{aligned} \varepsilon _{\infty \bot } & = \varepsilon _{\mathrm {d}}\dfrac{(1+f) \varepsilon _{\infty }+(1-f)\varepsilon _{\mathrm {d}}}{(1-f)\varepsilon _{\infty }+(1+f) \varepsilon _{\mathrm {d}}},\end{aligned}$$

20 $$\begin{aligned} F_{\bot } & = \dfrac{4f\varepsilon _{\mathrm {d}}}{(1-f)\left[ (1+f)\varepsilon _{\infty }+(1-f)\varepsilon _{\mathrm {d}}\right] },\end{aligned}$$

21 $$\begin{aligned} \omega _{\mathrm {p}\parallel }^{2} & = \dfrac{f\varepsilon _{\infty }\omega _{\mathrm {p}0}^{2}}{f\varepsilon _ {\infty }+(1-f)\varepsilon _{\mathrm {d}}},\end{aligned}$$

22 $$\begin{aligned} \omega _{0\bot }^{2} & = \dfrac{(1-f)\varepsilon _{\infty }\omega _ {\mathrm {p}0}^{2}}{(1-f)\varepsilon _{\infty }+(1+f)\varepsilon _{\mathrm {d}}},\end{aligned}$$

23 $$\begin{aligned} \omega _{0\parallel } & = 0. \end{aligned}$$

These equations indicate that the optical responses of a nanowire HMM in directions perpendicular to the optical axis and parallel to it are similar to the Lorentz and Drude media, respectively, as shown in Fig. 1b. Using the above equations, we can obtain the energy relations of a nanowire HMM according to the Eqs. (9) and (11). For the case $f\ll 1$, nanowires are well separated by large distances and there is no intermodal coupling between eigenmodes of them. Then from Eqs. (18)–(22) we find:

$$\begin{aligned} \varepsilon _{\infty \parallel }=\varepsilon _{\mathrm {d}} ,\;\;\;\;\varepsilon _{\infty \bot }=\varepsilon _{\mathrm {d}} , \end{aligned}$$

24 $$\begin{aligned} \omega _{\mathrm {p}\parallel }^{2}=\dfrac{f\varepsilon _{\infty }\omega _{\mathrm {p}0}^{2}}{\varepsilon _{\mathrm {d}}},\;\;\;\;\; \omega _{0\bot }^{2}=\dfrac{\varepsilon _{\infty }\omega _{\mathrm {p}0}^{2}}{\varepsilon _{\infty } +\varepsilon _{\mathrm {d}}},\;\;\;\;F_{\bot }=\dfrac{4f\varepsilon _{\mathrm {d}}}{ \varepsilon _{\infty }+\varepsilon _{\mathrm {d}}}, \end{aligned}$$

where $\sqrt{\varepsilon _{\infty }}\omega _{\mathrm {p}0}/\sqrt{\varepsilon _{\infty }+\varepsilon _{\mathrm {d}}}$ is the frequency of dipolar resonance of a single metallic nanowire surrounded by a dielectric medium^43^. Note that for type-I HMM we should have $\mathrm{Re}\left[ \varepsilon _{\mathrm {\parallel }}\right] <0$ and $\mathrm{Re}\left[ \varepsilon _{\mathrm {\bot }}\right] >0$ (${\text {Re}}$ means real part) and the imaginary parts of them must be small enough to be negligible. Therefore, using Eq. (3) we find $\mathrm{Re}\left[ \varepsilon _{\mathrm {\parallel }}\right] <0$, if $\omega <\sqrt{\omega _{\mathrm {p}\parallel }^{2}-\gamma ^{2}}$, and $\mathrm{Re}\left[ \varepsilon _{\mathrm {\bot }}\right] >0$, if $\omega <\omega _{0\bot }$, that means we may have $\omega <\mathrm {min}\left( \sqrt{\omega _{\mathrm {p}\parallel }^{2}-\gamma ^{2}},\omega _{0\bot }\right)$. Although the results in this subsection were derived for a type-I nanowire HMM, however, the expressions are generally valid and can be used for a type-II nanowire HMM (in an appropriate frequency region). It is easy to find that for type-II nanowire HMM we may have $\mathrm {max}\left( \sqrt{\omega _{\mathrm {p}\parallel }^{2}-\gamma ^{2}},\omega _{0\bot }\right)<\omega <\sqrt{1+F_{\bot}}\omega _{0\bot}.$

Now, we study the corresponding dispersion/anisotropy and absorption effects in the energy density and power loss. Therefore, we use the directionality factors, as ${\mathcal {D}}_{\mathrm {energy}}=\Xi _{\bot }/\Xi _{\parallel }$, where $\Xi _{\parallel }$ and $\Xi _{\bot }$ are the effective energy coefficients in Eq. (9) as mentioned before, and ${\mathcal {D}}_{\mathrm {loss}}=\Gamma _{\bot }/\Gamma _{\parallel }$, where $\Gamma _{\parallel }$ and $\Gamma _{\bot }$ are the effective energy loss coefficients in Eq. (11). We assume $\varepsilon _{\mathrm {d}}=\varepsilon _{\mathrm {SiO_{2}}=3.9}$, $\varepsilon _{\infty }=1$, and $\gamma =0.01\omega _{\mathrm {p}0}$.

Figure 3 shows the variation of ${\mathcal {D}}_{\mathrm {energy}}$ (blue curves), and ${\mathcal {D}}_{\mathrm {loss}}$ (red curves) as a function of the dimensionless frequency $\omega /\omega _{\mathrm {p}0}$ for a nanowire HMM as type-I HHM (for example a gold nanowire HMM in the visible and near-IR range^28^) with different values of *f*. The energy storage ability and the absorption property of the system can be isotropic with respect to the electric field for specific cases. For example, from panel (c) one can see the absorption property becomes isotropic when $f=0.2$ and $\omega =\sqrt{\omega _{\mathrm {p}\parallel }^{2} -\gamma ^{2}}=0.245\omega _{\mathrm {p}0}$. Panels (c) and (d) show that the electric field should be applied to the direction normal to the optical axis to store more energy in the medium, when the frequency is in the vicinity of $\sqrt{\omega _{\mathrm {p}\parallel }^{2}-\gamma ^{2}}$. Also, we see ${\mathcal {D}}_{\mathrm {loss}}$ and ${\mathcal {D}}_{\mathrm {energy}}$ decrease with decreasing the value of $\omega /\omega _{\mathrm {p}0}$. It is easy to find that the energy storage ability of the system corresponding to low values of $\omega$ is high, although the directionality factor indicates that the energy storage ability of the medium is anisotropic. Actually, for low values of $\omega$, the electric field should be applied to the direction parallel to the optical axis to store more energy in the medium.

### Multilayer hyperbolic metamaterials

Consider now a multilayer structure consisting of isotropic metal/dielectric layers, as shown in Fig. 2a. The *z*-axis is along the optical axis. Let $\varepsilon _{\mathrm {m}}$ ($\varepsilon _{\mathrm {d}}$) be the relative permittivity of the metal (dielectric) layer, and let *f* be the filling ratio of the metal layer satisfying $0<f<1$. Again, Eq. (17) shows the relative permittivity of a metallic layer. This multilayer structure can be used to construct an electric HMM of type II (in an appropriate frequency region) with the components of effective permittivity tensor given by^21,44^

25 $$\begin{aligned} \varepsilon _{\mathrm {\parallel }} & = \dfrac{\varepsilon _{\mathrm {m}} \varepsilon _{\mathrm {d}}}{(1-f)\varepsilon _{\mathrm {m}} +f\varepsilon _{\mathrm {d}}},\;\;\;\;\;(>0), \end{aligned}$$

26 $$\begin{aligned} \varepsilon _{\mathrm {\bot }} & = f\varepsilon _{\mathrm {m}} +(1-f)\varepsilon _{\mathrm {d}},\;\;\;\;\;\;(<0). \end{aligned}$$

We can rewrite Eqs. (25) and (26) as Eq. (3) [see the recipe for reducing Eqs. (15) and (16) to Eq. (3)]. In this case, we find

**Figure 4:**
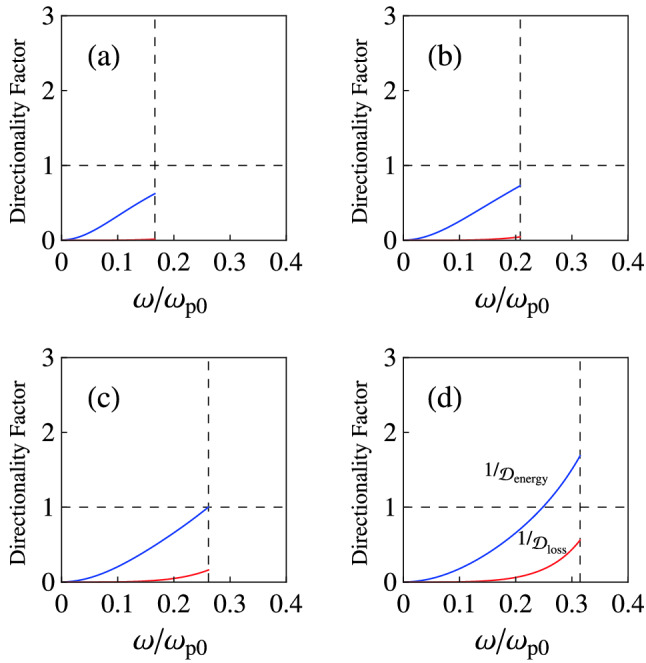
Variation of $1/{\mathcal {D}}_{\mathrm {energy}}$ (blue curves), and $1/{\mathcal {D}}_{\mathrm {loss}}$ (red curves) as a function of the dimensionless frequency $\omega /\omega _{\mathrm {p}0}$ for a multilayer HMM as type-II HHM (for example gold-SiO$_{2}$ multilayer HMM in the visible and near-IR range^28^). Here, the permittivity $\varepsilon _{\mathrm {m}}$ of a gold layer is characterized by the Drude model and we use $\varepsilon _{\mathrm {d}}=\varepsilon _{\mathrm {SiO_{2}}}=3.9$. For the other parameters we consider $\varepsilon _{\infty }=1$, and $\gamma =0.01\omega _{\mathrm {p}0}$. The vertical dashed line shows $\omega =\sqrt{\omega _{\mathrm {p}\bot }^{2} -\gamma ^{2}}$. The different panels refer to (**a**) $f=0.1$, (**b**) $f=0.15$, (**c**) $f=0.22{3}$, and (**d**) $f=0.3$.

27 $$\begin{aligned} \varepsilon _{\infty \bot } & = f\varepsilon _{\infty }+(1-f)\varepsilon _{\mathrm {d}}, \end{aligned}$$

28 $$\begin{aligned} \varepsilon _{\infty \parallel } & = \dfrac{\varepsilon _{\infty }\varepsilon _{\mathrm {d}}}{(1-f)\varepsilon _{\infty }+f\varepsilon _{\mathrm {d}}},\end{aligned}$$

29 $$\begin{aligned} F_{\parallel } & = \dfrac{f}{1-f}\dfrac{\varepsilon _{\mathrm {d}}}{\varepsilon _{\infty }},\end{aligned}$$

30 $$\begin{aligned} \omega _{\mathrm {p}\bot }^{2} & = \dfrac{f\varepsilon _{\infty }\omega _{\mathrm {p}0}^{2}}{f\varepsilon _{\infty }+(1-f)\varepsilon _{\mathrm {d}}},\end{aligned}$$

31 $$\begin{aligned} \omega _{0\parallel }^{2} & = \dfrac{(1-f)\varepsilon _ {\infty }\omega _{\mathrm {p}0}^{2}}{(1-f)\varepsilon _{\infty }+f\varepsilon _{\mathrm {d}}},\end{aligned}$$

32 $$\begin{aligned} \omega _{0\bot } & = 0. \end{aligned}$$

These equations show that the optical responses of a multilayer HMM in directions perpendicular to the optical axis and parallel to it are similar to the Drude and Lorentz media, respectively, as shown in Fig. 2b. Using the above equations, we can obtain energy relations of a multilayer HMM according to the Eqs. (9) and (11). Again, if we consider $f\ll 1$ then from Eqs. (27)–(31) we find:

$$\begin{aligned} \varepsilon _{\infty \bot }=\varepsilon _{\mathrm {d}} ,\;\;\;\;\varepsilon _{\infty \parallel }=\varepsilon _{\mathrm {d}} , \end{aligned}$$

33 $$\begin{aligned} \omega _{\mathrm {p}\bot }^{2}=\dfrac{f\varepsilon _{\infty }\omega _{\mathrm {p}0}^{2}}{\varepsilon _{\mathrm {d}}},\;\;\;\;\;\omega _{0\parallel }^{2} =\omega _{\mathrm {p}0}^{2},\;\;\;\;\;F_{\parallel }=f\dfrac{\varepsilon _{\mathrm {d}}}{\varepsilon _{\infty }}, \end{aligned}$$

where $\omega _{\mathrm {p}0}$ is the resonance frequency of a single metallic layer in a dielectric medium. This result indicates that the resonance effect also happens in the metallic layers. Note that this resonance frequency is free from the effect of permittivity of the dielectric medium.

Also, note that for type-II HMM we should have $\mathrm{Re}\left[ \varepsilon _{\mathrm {\parallel }}\right] >0$ and $\mathrm{Re}\left[ \varepsilon _{\mathrm {\bot }}\right] <0$. Therefore, using Eq. (3) we find $\mathrm{Re}\left[ \varepsilon _{\parallel }\right] >0$, if $\omega <\omega _{0\parallel }$, and $\mathrm{Re}\left[ \varepsilon _{\bot }\right] <0$, if $\omega <\sqrt{\omega _{\mathrm {p}\bot }^{2}-\gamma ^{2}}$, that means $\omega <\mathrm {min}\left( \sqrt{\omega _{\mathrm {p}\bot }^{2}-\gamma ^{2}},\omega _{0\parallel }\right)$. Although the results in this subsection were derived for a type-II multilayer HMM, however, the expressions are generally valid and can be used for a type-I multilayer HMM (in an appropriate frequency region). It is easy to find that for type-I multilayer HMM we may have $\mathrm {max}\left( \sqrt{\omega _{\mathrm {p}\bot }^{2}-\gamma ^{2}},\omega _{0\parallel }\right)<\omega <\sqrt{1+F _{\parallel }}\omega _{0\parallel }$.

Let us again study the corresponding dispersion/anisotropy and absorption effects in the energy density and power loss, when $\varepsilon _{\mathrm {d}}=\varepsilon _{\mathrm {SiO_{2}}=3.9}$, $\varepsilon _{\infty }=1$, and $\gamma =0.01\omega _{\mathrm {p}0}$. Figure 4 shows the variation of $1/{\mathcal {D}}_{\mathrm {energy}}$ (blue curves), and $1/{\mathcal {D}}_{\mathrm {loss}}$ (red curves) as a function of the dimensionless frequency $\omega /\omega _{\mathrm {p}0}$ for a multilayer HMM as type-II HHM (for example gold-SiO$_{2}$ multilayer HMM in the visible and near-IR range^28^) with different values of *f*. As a specific case, one can see that the energy storage ability of the system becomes isotropic with respect to the electric field, when $f\approx 0.223$ and $\omega =\sqrt{\omega _{\mathrm {p}\bot }^{2}-\gamma ^{2}}=0.262\omega _{\mathrm {p}0}$. The power loss corresponding to these specific values is low, although the directionality factor indicates that the absorption property of the medium near $\omega =0.262\omega _{\mathrm {p}0}$ is also anisotropic. Below this frequency, the system can store more energy if the electric field direction is parallel to the layers (normal to the optical axis).

## Some applications

### Energy velocity of TM wave in hyperbolic metamaterials

As an example of the application of the general energy density expression shown by Eq. (9), we now derive the formula for the energy velocity of a propagating transverse magnetic (TM) wave in an HMM. We note that the hyperbolic feature appears only for the TM polarized waves. For the sake of convenience, we consider a TM polarized wave with the field components $E_{\bot }=E_{x}$, $E_{\parallel }=E_{z}$, and $H_{y}$ as

34 $$\begin{aligned} \mathbf{H }=H_{0}\mathbf{e }_{y}e^{i\left( k_{\parallel }z+k_{\bot }x-\omega t \right) }, \end{aligned}$$

35 $$\begin{aligned} \mathbf{E }=\dfrac{H_{0}}{\omega \varepsilon _{0}}\left( \dfrac{k_{\parallel }}{\varepsilon _{\bot }}\mathbf{e }_{x}-\dfrac{k_{\bot }}{\varepsilon _{\parallel }}\mathbf{e }_{z} \right) e^{i\left( k_{\parallel }z+k_{\bot }x-\omega t \right) }, \end{aligned}$$

where the amplitude $H_{0}$, has been defined to be real. Also, $\mathbf{e }_{x}$, $\mathbf{e }_{y}$ and $\mathbf{e }_{z}$ are the unit vectors along the *x*, *y* and *z* axis, respectively, and $k_{\bot }=k_{x}$, and $k_{\parallel }=k_{z}$ denote the *x*, and *z* components of the wavevector. Note that

**Figure 5:**
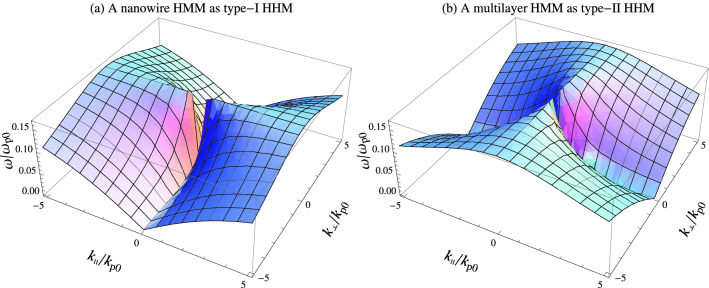
The dispersion relation for (**a**) a nanowire HMM (for example gold nanowire HMM), and (**b**) a multilayer HMM (for example gold-SiO$_{2}$ multilayer HMM) in the visible and near-IR range^28^, as type-I and type-II HHMs, respectively. Here, the permittivity of the metal (for example gold) $\varepsilon _{\mathrm {m}}$ is characterized by the Drude model and for the dielectric we use SiO$_{2}$ with $\varepsilon _{\mathrm {d}}=\varepsilon _{\mathrm {SiO_{2}}}=3.9$. For the other parameters we consider $\varepsilon _{\infty }=1$, $f=0.1$, and $\gamma =0$. Also, $k_{\mathrm {p}0}$ is the wavenumber corresponding to the electron plasma frequency $k_{\mathrm {p}0}=\omega _{\mathrm {p}0}/c$. The plots are symmetric about the planes $k_{\parallel }=0$ and $k_{\bot }=0$.

36 $$\begin{aligned} \dfrac{k_{\parallel }^{2}}{\varepsilon _{\bot }}+\dfrac{k_{\bot }^{2}}{\varepsilon _ {\parallel }}=\dfrac{\omega ^{2}}{c^{2}}, \end{aligned}$$

is the well-known dispersion relation of this TM wave^25^, where *c* is the speed of light in free space. The resulting dispersion relation is plotted in Fig. 5 for a nanowire HMM and a multilayer HMM as type-I and type-II HMMs in an appropriate frequency region. We assume that HMMs are nonabsorbing. In both cases, the contours of constant $\omega$ are hyperbolas. In the type-I HMM, these hyperbolas are centered on the $k_{\bot }$ axis, while in the type-II HMM, they are centered on the $k_{\parallel }$ axis^23,45^.

The exact definition of the velocity, $\mathbf{v }_{\mathrm {E}}$, with which energy is transported through the HMM is^46^

37 $$\begin{aligned} \mathbf{v }_{\mathrm {E}}=\dfrac{\mathbf{S }}{U}, \end{aligned}$$

where $\mathbf{S }$ is the time-averaged power flow and can be obtained from the Poynting vector by

38 $$\begin{aligned} \mathbf{S }=\dfrac{1}{2}{\text {Re}}\left[ \mathbf{E }\times \mathbf{H }^{*}\right] , \end{aligned}$$

in the complex number representation. This vector in the medium has components in the *x*- and *z*-directions, as

39 $$\begin{aligned} S_{\parallel }= & {} +\dfrac{1}{2}{\text {Re}}\left[ E_{\bot }H_{y}^{*}\right] =\dfrac{1}{2\omega \varepsilon _{0}} H_{0}^{2}{\text {Re}}\left[ \dfrac{k_{\parallel }}{\varepsilon _{\bot }} \right] , \end{aligned}$$

40 $$\begin{aligned} S_{\bot }= & {} -\dfrac{1}{2}{\text {Re}}\left[ E_{\parallel }H_{y}^{*}\right] =\dfrac{1}{2\omega \varepsilon _{0}} H_{0}^{2}{\text {Re}}\left[ \dfrac{k_{\bot }}{\varepsilon _{\parallel }} \right] , \end{aligned}$$

where, using Eq. (9) and Eqs. (39) and (40), we find the energy velocity components

41 $$\begin{aligned} v_{\mathrm {E}\parallel }= & {} \dfrac{S_{\parallel }}{U}=\dfrac{\dfrac{1}{2\omega \varepsilon _{0}} H_{0}^{2}{\text {Re}}\left[ \dfrac{k_{\parallel }}{\varepsilon _{\bot }} \right] }{\dfrac{1}{4}\left[ \varepsilon _{0}\Xi _{\parallel } \vert \mathbf{E }_{\parallel }\vert ^{2}+\varepsilon _{0}\Xi _{\bot } \vert \mathbf{E }_{\bot }\vert ^{2}+\mu _{0} \vert \mathbf{H }\vert ^{2}\right] }, \end{aligned}$$

42 $$\begin{aligned} v_{\mathrm {E}\bot }= & {} \dfrac{S_{\bot }}{U}=\dfrac{\dfrac{1}{2\omega \varepsilon _{0}} H_{0}^{2}{\text {Re}}\left[ \dfrac{k_{\bot }}{\varepsilon _{\parallel }} \right] }{\dfrac{1}{4}\left[ \varepsilon _{0}\Xi _{\parallel } \vert \mathbf{E }_{\parallel }\vert ^{2}+\varepsilon _{0}\Xi _{\bot } \vert \mathbf{E }_{\bot }\vert ^{2}+\mu _{0} \vert \mathbf{H }\vert ^{2}\right] }. \end{aligned}$$

If the losses are negligible, we obtain

43 $$\begin{aligned} v_{\mathrm {E}\parallel }= & {} \dfrac{2\omega k_{\parallel }/\varepsilon _{\bot } }{\left( \Xi _{\parallel }+\varepsilon _{\parallel }\right) \dfrac{k_{\bot }^{2}}{\varepsilon _{\parallel } ^{2}} +\left( \Xi _{\bot }+\varepsilon _{\bot }\right) \dfrac{k_{\parallel }^{2}}{\varepsilon _{\bot } ^{2}} }, \end{aligned}$$

44 $$\begin{aligned} v_{\mathrm {E}\bot }= & {} \dfrac{2\omega k_{\bot }/\varepsilon _{\parallel } }{\left( \Xi _{\parallel }+\varepsilon _{\parallel }\right) \dfrac{k_{\bot }^{2}}{\varepsilon _{\parallel } ^{2}} +\left( \Xi _{\bot }+\varepsilon _{\bot }\right) \dfrac{k_{\parallel }^{2}}{\varepsilon _{\bot } ^{2}} }, \end{aligned}$$

where these results for $\gamma =0$ can be deduced by taking the $\mathbf{k }$-gradient of the dispersion relation (36), i.e., $\mathbf{v }_{\mathrm {G}}=\nabla _{\mathbf{k }}\omega$. Therefore, we find the equality between the group velocity and the energy velocity, when $\gamma =0$. This equality is another verification of the presented results.

### Wave damping property

As another example of the application of the energy density expression, we now study the frequency dependence of the damping function of a propagating TM wave in an HMM. To obtain an analytical expression for the damping function (which depends on the frequency and wavenumber) of a propagating TM wave in an HMM, we use the perturbative method proposed by Loudon^40^. Such a procedure enables us to calculate the true wave damping rate to the first order in the damping parameter $\gamma$, introduced to describe the intrinsic damping of crystal oscillations. The advantage of perturbative method is that the damping properties result from the calculation of real dispersion relations. The plasmonic damping parameter or relaxation rate $\Gamma (k_{\parallel },k_{\bot },\omega )$ of the present case may be determined by the following procedure. The kinetic and total energy densities (per unit area) $U_{\mathrm {K}}$, and *U* are first calculated in the absence of damping. If a small amount of damping is now reintroduced, the wave relaxation rate to lowest order in $\gamma$ is

45 $$\begin{aligned} \Gamma (k_{\parallel },k_{\bot },\omega )=2\gamma \dfrac{U_{\mathrm {K}}}{U}, \end{aligned}$$

where from Eq. (14) we have

46 $$\begin{aligned} U_{\mathrm {K}}=\dfrac{\varepsilon _{0}}{4}\left[ \Gamma _{\parallel } \vert E_{\parallel }\vert ^{2}+\Gamma _{\bot } \vert E_{\bot }\vert ^{2}\right] , \end{aligned}$$

Therefore, we get

47 $$\begin{aligned} \Gamma (k_{\parallel },k_{\bot },\omega )= & {} 2\gamma \dfrac{\Gamma _{\parallel }\dfrac{k_{\bot }^{2}}{\varepsilon _{\parallel } ^{2}}+\Gamma _{\bot } \dfrac{k_{\parallel }^{2}}{\varepsilon _{\bot } ^{2}} }{\left( \Xi _{\parallel }+\varepsilon _{\parallel }\right) \dfrac{k_{\bot }^{2}}{\varepsilon _{\parallel } ^{2}} +\left( \Xi _{\bot }+\varepsilon _{\bot }\right) \dfrac{k_{\parallel }^{2}}{\varepsilon _{\bot } ^{2}} }. \end{aligned}$$

Let us note that the frequency dependence of the damping function comes from the retarded part of the plasmonc waves and one can find that, in the nonretarded limit, the total energy density becomes twice as large as the kinetic energy density of the system. As a consequence, the damping function of plasmonic waves of the system equals $\gamma$, i.e., it becomes a constant.

### Wave relaxation time

Let us note that the wave relaxation time $\mathcal {T}$, which determines the time rate of decay of power flow, is simply the inverse of Eq. (47), i.e.,

48 $$\begin{aligned} \mathcal {T}\left( k_{\parallel },k_{\bot },\omega \right) =\dfrac{1}{\Gamma \left( k_{\parallel },k_{\bot },\omega \right) }. \end{aligned}$$

In fact, $\mathcal {T}$ is the time at which the power flow at a point moving with the energy velocity is reduced to 1/*e* of its original value.

### Wave propagation length

The wave propagation length $\mathcal {L}$ is given by

49 $$\begin{aligned} \mathcal {L}\left( k_{\parallel },k_{\bot },\omega \right) =\dfrac{v_{\mathrm {E}}}{\Gamma \left( k_{\parallel },k_{\bot },\omega \right) }, \end{aligned}$$

where $v_{\mathrm {E}}$ can be found from Eqs. (43) and (44) as $v_{\mathrm {E}}=\left( v_{\mathrm {E}\parallel }^{2}+v_{\mathrm {E}\bot }^{2} \right) ^{1/2}$. In fact, the wave propagation length $\mathcal {L}$ is the distance after which the power flow in the wave is reduced to 1/*e* of its original value. Let us note that the absorption coefficient for the wave $\mathcal {A}$ is simply the inverse of Eq. (49), i.e.,

50 $$\begin{aligned} \mathcal {A}\left( k_{\parallel },k_{\bot },\omega \right) =\dfrac{1}{\mathcal {L}\left( k_{\parallel },k_{\bot },\omega \right) }. \end{aligned}$$

## Conclusion

In summary, it seems that the structure of an HMM is much simpler than the wire-SRR and chiral metamaterials. But, the effective permittivities of HMMs are more complex than those of wire-SRR and chiral metamaterials. Therefore, the investigation of electromagnetic energy density in such media may be difficult. To remedy this hardship, as a non-trivial and key step, we have obtained familiar forms for the well-known effective permittivities of nanowire HMMs and multilayer HMMs, similar to the Lorentz and Drude media. These new transparent forms for the effective permittivities of the HMMs simply show that HMMs have different dynamical properties in the directions parallel and perpendicular to their optical axes. In this way, we have extended the previous results for the electromagnetic energy density in the single-resonance chiral^18^ and the wire-SRR^11,17^ metamaterials, and simply derived the energy density associated with an electromagnetic wave passing through an HMM. Note that we have checked the validity of the obtained results in two steps. First, we have shown the time-averaged energy density formula derived here, i.e., Eq. (9) is consistent with the well-known Landau formula, i.e., Eq. (13), when the losses are negligible. Second, we have shown the group velocity of the TM waves in a lossless HMM is the same as the energy velocity (i.e., the ratio of the power flow to the storage energy). Here we should stress that in the present work, we mainly study the electromagnetic energy density of TM waves in the electric HMMs. Similar results can be obtained in the propagation of transverse electric (TE) waves in the magnetic HMM^47^.

## Supplementary Information

Supplementary Information.

## Data Availability

The data that supports the findings of this study are available within the article.
