# Supplementary material for: Electromagnetic energy density in hyperbolic metamaterials
Source: Sci Rep. 2022 Jun 24;12:10760. doi: 10.1038/s41598-022-14909-0 (PMC9232634; doi:10.1038/s41598-022-14909-0)
Supplement: Supplementary file 1 — Supplementary Information. [file 41598_2022_14909_MOESM1_ESM.pdf]

Supplementary Material: Electromagnetic Energy Density in Hyperbolic Metamaterials

Afshin Moradi[1] and Pi-Gang Luan[2]

[1]Department of Engineering Physics, Kermanshah University of Technology, Kermanshah, Iran

[2]Department of Optics and Photonics, National Central University, Jhongli District, Taoyuan City 320, Taiwan

Corresponding author: a.moradi@kut.ac.ir (A. Moradi)

Consider a composite of metallic nano-spheres with relative dielectric constant $\varepsilon_{\rm m}$ [see Eq. (17)] embedded in a host matrix with relative dielectric constant $\varepsilon_{\rm d}$. Let f be the volume fraction of the embedded nano-spheres satisfying $0 < f < 1$. For the relative effective permittivity of the present composite, we have [1, 2]

$$\frac{\varepsilon_{\rm eff} - \varepsilon_{\rm d}}{\varepsilon_{\rm eff} + 2\varepsilon_{\rm d}} = f \frac{\varepsilon_{\rm m} - \varepsilon_{\rm d}}{\varepsilon_{\rm m} + 2\varepsilon_{\rm d}} , \tag{A-1}$$

or

$$\varepsilon_{\rm eff} = \varepsilon_{\rm d} \frac{(\varepsilon_{\rm m} + 2\varepsilon_{\rm d}) + 2f(\varepsilon_{\rm m} - \varepsilon_{\rm d})}{(\varepsilon_{\rm m} + 2\varepsilon_{\rm d}) - f(\varepsilon_{\rm m} - \varepsilon_{\rm d})} , \quad (> 0) . \tag{A-2}$$

We can rewrite Eq. (A-2) [see the recipe for reducing Eq. (16) to Eq. (3)] as

$$\varepsilon_{\rm eff} = \varepsilon_{\rm b} \left(1 - \frac{F\omega_0^2}{\omega(\omega + i\gamma) - \omega_0^2} \right) , \tag{A-3}$$

where

$$\varepsilon_{\rm b} = \varepsilon_{\rm d} \frac{(\varepsilon_\infty + 2\varepsilon_{\rm d}) + 2f(\varepsilon_\infty - \varepsilon_{\rm d})}{(\varepsilon_\infty + 2\varepsilon_{\rm d}) - f(\varepsilon_\infty - \varepsilon_{\rm d})} , \tag{A-4}$$

$$F = \frac{9f\varepsilon_{\rm d}}{(1-f)\left[(\varepsilon_\infty + 2\varepsilon_{\rm d}) + 2f(\varepsilon_\infty - \varepsilon_{\rm d})\right]} , \tag{A-5}$$

$$\omega_0^2 = \frac{(1-f)\varepsilon_\infty \omega_{\rm p0}^2}{(\varepsilon_\infty + 2\varepsilon_{\rm d}) - f(\varepsilon_\infty - \varepsilon_{\rm d})} . \tag{A-6}$$

For the case $f \ll 1$ we find $\omega_0 = \varepsilon_\infty \omega_{\rm p0} / \sqrt{\varepsilon_\infty + 2\varepsilon_{\rm d}}$ that is the frequency of dipolar resonance of a single metallic nano-sphere surrounded by a dielectric medium [3]. Although Eq. (A-3) was derived for the case $\varepsilon_{\rm eff} > 0$, however, this expression is generally valid and can be used when $\varepsilon_{\rm eff} < 0$.

References

[1] U. Kreibig, and M. Vollmer, *Optical Properties of Metal Clusters*, (Springer, Berlin, 1995).

[2] A. Moradi, *Canonical Problems in the Theory of Plasmonics: From 3D to 2D Systems*, (Springer, Switzerland, 2020).

[3] A. Moradi, High-frequency waves in a random distribution of metallic nanoparticles in an external magnetic field, Z. Naturforsch. **71**, 849 (2016).
